# Friends or foes? The knowns and unknowns of natural killer cell biology in COVID-19 and other coronaviruses in July 2020

**DOI:** 10.1371/journal.ppat.1008820

**Published:** 2020-08-26

**Authors:** Cordelia Manickam, Sho Sugawara, R. Keith Reeves

**Affiliations:** 1 Center for Virology and Vaccine Research, Beth Israel Deaconess Medical Center, Harvard Medical School, Boston, Massachusetts, United States of America; 2 Ragon Institute of Massachusetts General Hospital, MIT, and Harvard, Cambridge, Massachusetts, United States of America; University of Alberta, CANADA

## Abstract

The COVID-19 pandemic has caused more than 575,000 deaths worldwide as of mid-July 2020 and still continues globally unabated. Immune dysfunction and cytokine storm complicate the disease, which in turn leads to the question of whether stimulation or suppression of the immune system would curb the disease. Given the varied antiviral and regulatory functions of natural killer (NK) cells, they could be potent and powerful immune allies in this global fight against COVID-19. Unfortunately, there is somewhat limited knowledge of the role of NK cells in SARS-CoV-2 infections and even in the related SARS-CoV-1 and MERS-CoV infections. Several NK cell therapeutic options already exist in the treatment of tumor and other viral diseases and could be repurposed against COVID-19. In this review, we describe the current understanding and potential roles of NK cells and other Fc receptor (FcR) effector cells in SARS-CoV-2 infection, advantages of using animals to model COVID-19, and NK cell–based therapeutics that are being investigated for COVID-19 therapy.

## NK cell background

**Fig 1:**
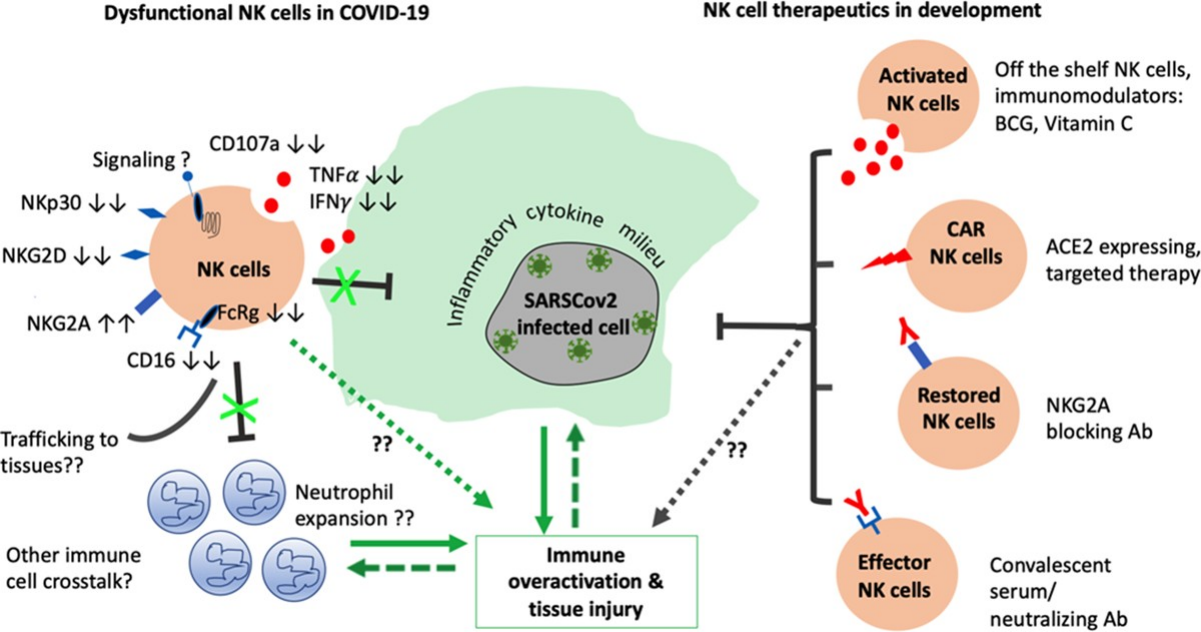
Potential roles of NK cells and NK cell–based interventions in COVID-19. FcRg, Fc receptor gamma chain; IFNγ, interferon-gamma; NK, natural killer; TNFα, tumor necrosis factor alpha. NK cells in COVID-19–infected people exhibit lower expression of activating receptors including NKp30, NKG2D, NKG2C, CD16, and Fc receptor γ chain (FcRγ) and higher inhibitory NKG2A expression. As a consequence, NK cells exert reduced degranulation and cytokine secretion, which could interactions with other immune cells and contribute to overall hyperimmune activation and tissue injury. Several NK cell–based therapeutics currently in development against COVID-19 infection employ different strategies, including inducing NK cell activation, inhibiting NK exhaustion, and eliciting effector functions of NK cells against infected cells for early clearance of viral infected cells, and prevent tissue injury.

Natural killer (NK) cells are innate lymphocytes that provide rapid and efficient responses against pathogens and tumors. NK cells are present in healthy lymphoid and mucosal tissues and are swiftly mobilized to sites of infection. The phenotype and functions of human NK cells can be complex and varied depending on the tissue, ranging from pathogen clearance by cytotoxic responses to maintenance of homeostasis by immune-regulation. Broadly, CD56^bright^ immature NK cells are secretors of proinflammatory cytokines, and CD56^dim^CD16^high^ mature NK cells are cytotoxic in function. NK cells are also used in development of novel biotherapeutics and vaccines due to their potent functions. However, there is a huge gap in the understanding of the roles and functions of NK cells in COVID-19 infection. In this review, we strive to provide an overview of what is known about NK cells in SARS-CoV-2 patients and animal models, and their potential roles in stand-alone or combination therapy against the pandemic (Fig 1).

## COVID-19 virology and pathogenesis

The novel SARSCoV2 is an enveloped, positive sense, single stranded RNA virus of the genus *Betacoronavirus* and family Coronaviridae, with more than 80% and 50% homology to SARS-CoV-1 and MERS-CoV, respectively [1,2,3,4]. While there are stills gaps in the understanding of the pathogenesis of SARS-CoV-2, the increasingly growing body of COVID research and the information available from the previous 2 coronaviral epidemics, SARS and MERS, has helped piece together the clinical course of COVID-19 infection [5,6], which Mason and colleagues [7] describe as 3 stages of infection. SARS-CoV-2, similar to SARS-CoV-1, primarily spreads through the intranasal route, in which the viral spike protein binds to the receptor angiotensin converting enzyme 2 (ACE2) that is expressed on many cells of the respiratory tract [8]. With a mean incubation period of 5.1 days, the first stage of COVID-19 infection is characterized by local viral replication and shedding in the upper respiratory tract even in the absence of clinical symptoms, thus making asymptomatic patients infectious and further facilitating easy spread of infection. In the second stage of infection, the virus spreads to lower respiratory tract at which time a robust innate immune response is elicited and is also marked by clinical disease of COVID-19. The most common symptoms of COVID-19 include fever, cough, and fatigue and, additionally, headache, dyspnea, hemoptysis, and diarrhea [9,10]. High blood neutrophil count particularly neutrophil to lymphocyte ratio (NLR), lymphopenia, and elevated proinflammatory cytokines and chemokines that include CCL7, interleukin (IL) -1β, IL-1RA, IL-7, IL-8, IL-9, IL-10, basic fibroblast growth factor 2 (FGF2), granulocyte colony-stimulating factor (G-CSF), granulocyte monocyte colony stimulation factor (GM-CSF), interferon (IFN)-γ, IP-10/CXCL10, monocyte chemoattractant protein (MCP)-1, monocyte inhibitory protein (MIP)-1α, MIP-1β, platelet derived growth factor subunit B (PDGFB), tumor necrosis factor (TNF)-α, and vascular endothelial factor (VEGF)-A in the plasma of COVID-19 patients have been reported [11–13].

Approximately 80% of infected patients manifest mild symptoms and are able to clear the virus. However, 20% of the infected patients progress to stage 3, which involves viral replication in the alveolar type II cells, pulmonary infiltration of immune cells leading to pathologic changes associated with severe pneumonia, and acute respiratory distress syndrome (ARDS) with fatal outcome [14]. Widespread damages in the lungs include pulmonary edema, diffuse alveolar damage with hyaline membranes, fibrosis, multinucleated giant cells, and cytopathic changes of the pneumocyte. Uncontrolled cytokine storm, as a product of the infiltrating immune cells in the lungs, causes severe systemic inflammation, ARDS, multiorgan failure, and death [14]. Further, fatal complications are particularly high in certain risk groups, such as the elderly, in whom the case fatality rate can be as high as 14.8% to 20% due to diminished protective immune responses and regenerative ability of the epithelium [15]. Similarly, the risk of complications in COVID-19 patients are significantly elevated in the presence of comorbidities that include diabetes, hypertension, chronic obstructive pulmonary disease, asthma, cardiovascular disease, and cancer [16].

The central immunopathogenic mechanism in COVID-19 associated complications and morbidities is the cytokine syndrome elicited by the unbridled inflammatory immune cells. Indeed, elevated IL-6, TNF-α, chemokine (C-C motif) ligand 7 (CCL7), and chemokine (C-X-C motif) ligand 10 (CXCL10) correlated with severity of disease [12] and negatively correlated with T cell counts [17,18]. Activated T helper 1 (Th1) cells expressing IFN-γ, IL-6, and GM-CSF and inflammatory CD14^+^CD16^+^ monocytes secreting IL-6 and GM-CSF have been reported in COVID-19 patients with severe pneumonia [19]. While there is limited data available on the role of NK cells in COVID-19 pathogenesis, particularly in lung pathology, the functional exhaustion of NK cells and T cells could further complicate the immune dysfunction in COVID-19 patients [18,20].

## Animal models for COVID-19

Given the zoonotic nature of SARS-CoV-2, it is unsurprising that several species including mice, ferrets, cats, and nonhuman primates (NHP) have been found to be susceptible to this virus [21]. Dogs have low susceptibility, while pigs, chickens, and ducks appear not to be susceptible to SARS-CoV-2 [21]. Animal models including mice, Syrian hamsters, and NHP have been useful for understanding SARS and MERS previously and, therefore, could be useful to fill in gaps in our COVID-19 knowledge, such as immune correlates of protection, role of innate immunity in immune protection/ tissue pathology, antibody-based control of reinfections, and others. However, it is important to consider the strengths and weaknesses of the animal models in order to ascertain their suitability to model COVID-19, particularly for the rapid development of vaccine and therapeutic efficacy. For example, mice models were shown to be only semipermissive to SARS-CoV-1 infection since murine ACE2 binds less efficiently than human ACE2 to SARS-CoV-1 [22]. Similarly, SARS-CoV-2 failed to infect lungs of wild type mice [23]. The poor viral tropism was overcome by K18-hACE2 transgenic mice expressing human ACE2, which showed severe lung pathology with immune cell infiltration and up-regulated cytokine expression in the lungs [24]. However, this model delivered an altered clinical and fatal disease with 100% mortality and viral infiltration in the brain of SARS-CoV-1–infected mice [24], but no viral RNA or pathogenicity was observed in brains of SARS-CoV-2–infected mice [23]. While ferrets are susceptible to SARSCoV2, only mild clinical disease with high viral titers in upper respiratory tract and relatively lower viral titers in lungs was reported [25]. Shi and colleagues [26] also showed the presence of virus in nasal turbinate, soft palate, and tonsils but not in lungs, intestines, the brain, and other tissues, indicating that ferrets show only partial recapitulation of human disease.

Old World monkeys and humans have highly conserved ACE2 protein, with 100% identity for critical residues at the binding sites of ACE2 to SARS-CoV-2 [27]. The similar binding affinity of ACE2 in NHP allows them to be more permissive for viral replication. In addition, NHP are more comparable to humans in terms of lung anatomy, physiology, and immunology than rodent models [28]. SARS-CoV2–infected rhesus and cynomolgus macaques exhibited viral shedding in nasopharyngeal and oral samples, pneumonia and lung pathology characterized by diffuse alveolar damage (DAD), pulmonary edema and hyaline membrane formation, and accumulation of neutrophils, macrophages, and lymphocytes similar to COVID-19 patients [29–31]. Prolonged viral shedding and severe interstitial pneumonia was observed in aged macaques but did not accompany changes in disease outcome compared to young adults [30,32]. Neutralizing antibody development and cellular immunity after primary infection in rhesus macaques was found to protect against reinfection [14,31]. Similarly, an inactivated SARS-CoV-2 vaccine was found to induce neutralizing antibody development in macaques in addition to mice and rats and was capable of inducing complete protection against challenge [14]. Overall, these studies show that animal models, particularly NHP, can recapitulate human viral shedding, antiviral immune responses, and lung pathology; however, more animal studies with large sample size are essential to further elucidate COVID-19 pathogenesis and effectively test vaccines and therapeutic interventions.

## NK cells in COVID-19 disease

The role of NK cells in coronaviral infections has been mostly understudied because mice studies showed that NK cells may not be essential for viral clearance in SARS-CoV-1 and MERS-CoV infections. However, the recent COVID-19 vaccine study demonstrated the antispike dependent NK cell responses in vaccinated macaques [33], which indicates NK cells do exert functions towards COVID-19–infected cells. Nonetheless, most studies have not considered the alternate hypothesis that NK cells could in fact be pathologic and contribute to the pathogenesis in COVID-19 (Fig 1). In SARS-CoV-2–infected patients, the frequency of peripheral NK cells were reduced [17,18,20,34–37] or not significantly altered [13, 38]. Reduction in NK cell frequencies and their CD16 expression in severe COVID-19 patients reverted back to normal levels after recovery [37]. Functionally, peripheral NK cells and CD8 T cells exhibited reduced expression of CD107a, IFN-γ, and TNF-α in COVID-19 patients [39]. Elevated NKG2A expression on both NK and T cells indicative of exhaustion in the patients was restored to normal levels after therapy [39]. This upregulated NKG2A expression on NK cells was found to be mediated by the viral spike protein. In vitro coculture of peripheral NK cells with SARS-CoV-2 spike protein transfected lung epithelial cells resulted in reduced NK degranulation and up-regulation of NKG2A [34]. The concomitant histocompatibility leukocyte antigen E (HLA-E) expression on the lung epithelial cells shows that the spike protein could lead to NK cell exhaustion through HLA-E and NKG2A interaction [34]. The commentary by Antonioli and colleagues [40] suggests that this NKG2A expression on NK cells and T cells could be a critical factor in the immune dysfunction of COVID-19 patients, disrupting the crosstalk between these immune cells and neutrophils. The IFN-γ secreted by activated NK cells not only functions as an antiviral cytokine but is also important for the impairment of neutrophil expansion and survival in the lungs of TB-infected mice, thus regulating and limiting neutrophil mediated tissue injury [41]. The increased NKG2A expression on NK cells provides inhibitory signals, which in turn inhibits the NK cell expansion, while the neutrophil numbers remain uncurbed. This is further aggravated by the inflammatory milieu set up by the cytokine release syndrome, particularly IL-6 and IL-10, which have been shown to induce NKG2A expression [42] while inducing neutrophilia [43]. This phenomenon could be why COVID-19 patients have reduced frequencies of NK cells, T cells, and total lymphocyte counts, while a high NLR is one of the prognostic markers of poor disease outcome [44].

In a study comparing patients with COVID-19 pneumonia, non–COVID-19 pneumonia and healthy controls, Varchetta and colleagues [45] reported increased CD57+FcεRIγ- adaptive NK cells and CD16^+^ NK cells and decreased CD56^bright^ NK cells. While they did not observe any differences in NKG2A/C expression, there was reduced expression of Siglec-7, NKG2D, DNAM-1, and NKp30 in COVID-19 patients [45]. Interestingly, NK cells from these patients exerted more antibody dependent cell mediated cytotoxicity (ADCC) than healthy controls [45]. This study though limited by a small sample size, provides promising data that there is a potential development of memory-like or trained NK cells, as evidenced by the elevated FcεRIγ^-^ NK cells and improved ADCC response in COVID-19 patients. Similarly, Maucourant and colleagues [46] reported strong NK cell activation with expansion of adaptive NKG2C^+^ NK cell in the blood of COVID-19 patients. However, the high expression of NKG2C, perforin, and ksp (killer specific secretory protein)-37 correlated with disease severity. Perforin expression of CD56^bright^ NK cells positively correlated with IL-6, Sequential Organ Failure Assessment (SOFA) scores and neutrophil count and inversely correlated with partial pressure of oxygen (PaO2) and fraction of inspired oxygen (FiO2) in COVID-19 patients [46], indicating hyperinflammed state of NK cells in severe COVID-19. Granzyme A–expressing NK cells were negatively correlated with serum IL-6 levels in COVID-19 patients, and the impaired cytotoxic potential was restored when treated with tocilizumab [47]. These studies further emphasize the need for more rigorous studies with larger sample sizes to understand NK cells, which could prove useful in the current race against the COVID-19 pandemic since the virus mediated immune dysfunction appears to extend to NK cells [48]. More significantly, the role of NK cell at primary sites of infection, lungs, and other tissue would be more crucial to understand pathogenesis of this disease, since most studies in COVID-19 patients have been focused on immune cell subsets in the periphery.

## NK cells at sites of SARS-CoV-2 infection

Lung NK cells account for approximately 10% to 20% of lymphocytes in the parenchyma of human and murine lungs [49,50]. Of all nonlymphoid organs, lungs are the most enriched tissues in conventional NK cells, which suggests that they play major roles in homeostasis and infections. The lung NK subsets, composed of highly differentiated and mature NK cells that include CD56^dim^CD16^+^ and CD57^+^NKG2A^-^ NK cells than commonly observed in peripheral blood mononuclear cells (PBMC) and other tissues [49,50], were found to be hypofunctional in homeostasis, potentially due to the suppressive local microenvironment contributed by alveolar macrophages and the epithelial lining [51], thus tightly regulating pulmonary homeostasis. When homeostasis is disrupted during infections, the lung NK cells could have dichotomous roles, either beneficial leading to clearance of pathogen and/or deleterious causing tissue damage due to hyperresponsiveness. In influenza A virus (IAV)-infected mice models, activated lung NK cells clear virus through cytotoxicity, secretion of IFN-γ, and stimulation of adaptive cells [52]. In addition, IL-22-producing NKp46^+^ NK cells facilitating epithelial regeneration were found in bronchoalveolar lavage fluid (BALF), trachea, and lung tissues [53]. Conversely, depletion of NK cells alleviated lung immunopathology in high dose infected mice [54]. These studies suggest that the hyporesponsive lung NK cells are activated in respiratory infections and could lead to viral clearance and/or lung pathology, depending on the dose of the virus infection. In SARS-CoV-1 infected mice, immune cells including NK cells, NKT cells, macrophages, and CD4 T cells were elevated in lungs at day 2 postinfection and then declined after reaching a peak in numbers at day 7 [55]. There are very few studies investigating NK cells in mice models for SARS and MERS, and they have mostly shown that NK cells and their ADCC functions are not essential for viral clearance in the lungs [56–58]. However, rodent respiratory system do not recapitulate human lung immunology efficiently. NHP models have shown infiltration of immune cells and associated lung pathology [29,30,32], but these studies have not investigated lung NK cells. Overall, lung NK cells in COVID-19 have been mostly overlooked and warrant a closer look at their potential antiviral immune defenses.

The main SARS-CoV-2 entry receptor, ACE2, is also found in several tissues associated with the gastrointestinal (GI) tract, cardiovascular system, and brain. ACE2 is particularly enriched in the gut, where it is involved in regulation of intestinal amino acid transporters and thereby influences gut microbiome composition and intestinal inflammation [59]. Therefore, it is unsurprising that GI symptoms, such as nausea, vomiting, and diarrhea, are also associated with SARS-CoV-2 infection with viral shedding in fecal samples [60, 61]. NK cells are present in large and small intestine and localized in the intraepithelial lymphocyte compartment, lamina propria, and Peyer’s patches, and play important complex roles in regulating the gut microbiome, immune crosstalk, and immune defenses against gut pathogens. Unlike lung NK cells, the gut mucosal NK cells are predominantly immature NK cells of phenotype CD3^-^CD56^bright^CD16^-^ cells, which are primarily IFN-γ secreting and not cytotoxic [62]. While alterations in gut NK cell subsets have been reported in mucosal infections, particularly in HIV infection, there are no studies to provide information on coronavirus-mediated immunity or pathology in the gut of humans and animal models.

Neurologic manifestations including taste, smell, and/or vison impairment, acute cerebrovascular diseases, and impaired consciousness are increasingly reported in COVID-19 patients, particularly those with severe respiratory involvement and lymphocytopenia [63–66]. It is not yet clear if the neurological symptoms are due to the posited cytokine syndrome or the direct CNS invasion of the SARS-CoV-2 virus. In transgenic mice expressing human ACE2, SARS-CoV-1 entered the brain through the olfactory nerve, rapidly replicated in the brain, and up-regulated proinflammatory cytokines (including IL-6), but, surprisingly, there was no evidence of inflammation in the brain [67]. The fatal outcome of brain infection in this model could be attributed to any of these several mechanisms: neuronal infection and dropout, infection of the cardiorespiratory center in the medulla, and cytokine storm. NK cells are one of the key immune cells that can cross the blood brain barrier under pathological conditions. Indeed, NK cells were the major immune cells elevated in patients with disrupted blood brain barrier and were correlated with multiple proinflammatory cytokines and chemokines, such as IFN-γ, TNF-α, CCL3, and IL-6, in the cerebrospinal fluid of patients with neuroinflammation [68]. In mice infected with a recombinant murine coronavirus, mouse hepatitis virus (MHV) expressing CXCL10, showed CXCL10 mediated recruitment of NK cells into the brain and was critical in controlling MHV infection before T cell responses were activated in the brain [69]. These studies indicate the NK cells are recruited in the CNS during inflammation and could have potential neuroprotective roles against infections.

Limited NK cell research exists in other major organs such as kidneys, heart, and others [70,71], but they show that NK cells play substantial roles in both immune protection and/or tissue injury in the inflammatory pathogenesis of renal and cardiovascular diseases. While the peripheral immune responses and parameters are useful in generating disease outcome predictors for COVID-19, knowledge of immune cell responses at the local and mucosal tissue microenvironment are even more critical for viral control and limiting severity of disease. The lack of NK cell knowledge and in general early immune responses at the primary site of infection and the lungs in coronaviral infections in both humans and animal models is a huge roadblock in the path to control of the COVID-19 pandemic.

## Opportunities for NK cell–based intervention in COVID-19

Since the outbreak of COVID-19 infections worldwide began around January 2020, there has been a tremendous concerted effort to fast track therapeutics and vaccine development against SARS-CoV-2. Targeted cell therapy, bioengineered and vectored antibodies, and nucleic acid-based therapies are among some of the current approaches toward SARS-CoV-2. Already available biologics targeting other RNA viruses (such as SARS-CoV, MERS-CoV, HIV, and Ebola) and anti-inflammatory drugs to combat cytokine dysregulation are being repurposed against SARS-CoV-2 and provide the advantage of overcoming the lengthy and arduous process of new drug development. In this section we discuss some of the therapeutics and vaccine strategies that harness the rapid antitumor and antiviral functions of NK cells by employing strategies that range from boosting NK cell expansion and function in the body to NK cell reprogramming to confer them with memory, memory-like functions, and antigen targeting.

Activation and expansion of NK cells, particularly in early stages of infection, could prove useful against COVID-19 since an early viral clearance would limit the development of cytokine storm and ARDS (Fig 1). This can be achieved by the use of adjuvants and vaccines that modulate the innate response. For example, a few groups have hypothesized the role of BCG vaccination in the reduction of COVID-19 infections and mortality [72–74]. The hypothesis is based on observational reports built on COVID-19 data from countries with or without BCG vaccination. While these reports and this model have several limitations due to the variations in the reporting of case numbers, population densities, cultural differences, and others, the immunomodulatory effect of BCG vaccination in providing trained immunity is not mere speculation but rather well established. The BCG vaccine induces epigenetically modified and trained monocytes, macrophages, and NK cells with enhanced defensive functions against non-mycobacterial infections. Additionally, NK cells are essential for successful BCG immunotherapy [75,76] and therefore could prove beneficial against COVID-19. Clinical studies are underway in the Netherlands and Australia (ClinicalTrials.gov Identifier: NCT04327206 and NCT04328441) to test the efficacy of BCG vaccine in COVID-19 patients [76]. Similarly, vitamin C could also be used as an adjunct therapy since it promoted the ex vivo expansion of NK cells more than 10-fold while their tumor killing functions remained the same under normoxia and hypoxia [77]. A clinical trial to study the efficacy of vitamin C infusion for COVID-19 patients with severe pneumonia is also underway in China [78] (NCT04264533).

Functional exhaustion of NK cells and T cells seen in COVID-19 patients [39] could be targeted for restoration by blockade of exhaustion and inhibitory markers. Overexpression of NKG2A on NK and T cells leads to inhibitory signaling and suppressed cytotoxic functions and could further disrupt the NLR [40]. Monalizumab [79], which blocks NKG2A, has already cleared Phase II clinical trials against a number of cancers and could be a potential therapeutic candidate that could restore antiviral functions of NK and T cells and restore the NLR. However, it is important to point out that there is a risk of overactivation of NK cells, which could also result in tissue injury.

Chimeric antigen receptor (CAR) expressing NK and T cells have been effectively used as targeted cell therapy against tumors [80,81]. Recently, CAR-NK cells have been shown to overcome the life-threatening toxicities due to the cytokine release storm (CRS) that is commonly associated with CAR T cell therapy [81,82]. Peak IL-6 levels observed in the CRS associated with CAR T cell therapy was not observed with CAR-NK cells. Since IL-6 is the central cytokine in COVID-19 pathology as well, CAR-NK cells could provide antiviral responses without inducing the same inflammatory cytokines. CAR-NK cells co-expressing the SARS-CoV-2 entry receptor ACE2 and the activating NK receptor NKG2D are currently in clinical trials (NCT04324996). Their ACE2 expression specifically targets the spike protein of the virus and so can competitively inhibit virus attachment to host cells as well as destroy virus infected cells. The NKG2D receptor activation is mediated through NKG2D ligands expressed by infected cells. These CAR-NK cells are inclusive of additional enforcements such as IL-15 secretion for their long-term survival and persistence, and GM-CSF neutralizing scFv region to prevent CRS. Even with these protective armors, the potential risk of CAR-NK cells causing tissue damage is high [83]. Robust targeted killing of virus infected cells, which would include pneumocytes in the lungs and enterocytes in the intestine, without their replacement by functional counterparts could lead to scarring, fibrosis, and reduced organ function.

The usual lack of antigen specificity of NK cells, unlike T cells, can be exploited for therapeutic purposes. Two allogenic, off the shelf NK cell therapy are in clinical trials, of which one is derived from the blood of healthy donors (NCT04344548). The other, CYNK-001, developed by Celularity is derived from in vitro–expanded human placental CD34+ cells and is being tested for the treatment acute myeloid leukemia and multiple myeloma. Recently, the Food and Drug Administration (FDA) has approved the testing of CYNK-001 as a potential universal NK therapy for COVID-19 patients and is recruiting for Phase I and II trials (NCT04365101). While this NK cell therapy could be useful in killing virus infected cells and, thus, limit virus mediated inflammation, there is no publicly available data yet to substantiate projected therapeutic potential.

Important COVID-19 therapeutic approaches include convalescent serum and neutralizing antibody for thwarting viral infections. Several biotech/pharmaceutical companies, including Regeneron, AbCellera and Lily, Vir and GlaxoSmithKline, CytoDyn, AstraZeneca, and Amgen, are developing monoclonal antibody technologies against SARS-CoV-2 [84]. In addition to their roles in viral neutralization, the antibody-based therapeutics could also mediate effector functions by recruiting NK cells and other FcR-bearing cells for ADCC and ADCP. A study in mice infected with SARS-CoV-1 [58] showed that phagocytosis by macrophages was the major causative factor in viral clearance from lungs. Leronlimab that targets chemokine receptor CCR5 was originally developed to block HIV binding and breast cancer metastasis and is now being tested for COVID-19 therapy (NCT04347239). A significant risk with antibody therapy is the potential to develop antibody enhanced disease (ADE) as seen in dengue and vaccine induced ADE in RSV and atypical measles (reviewed in [85]). While, our current knowledge in human patients and animal models have shown no evidence of ADE and, therefore, could prove to be a low risk, more rigorous testing and experimental data is essential to understand the true risk posed by antibody-based therapeutics.

NK cell–based therapies for COVID-19 as stand-alone or combination therapy are very promising. However, as explained by Rajaram and colleagues [83], NK cell therapy could be associated with significant risks. There are several concerns that need to be addressed, such as overactivation of NK cells and secretion of inflammatory cytokines, destruction of pneumocytes, and tissue injury, which would perpetuate the ARDS and fatal outcomes associated with COVID-19 pathology. Parameters such as the timing of the NK cell therapy and the severity of the disease status need to be considered to tip the balance towards therapeutic protection. NK cell responses would be more effective early in the infection and would prevent progression of the disease. Immunomodulation of NK cells towards regulatory function could be useful as adjunct therapy in progressive patients. For example, IFN-β therapy induces regulatory NK cells that could control overt T cells responses [86]. A randomized Phase II trial in Hong Kong showed viral clearance in patients with mild and moderate COVID-19 when treated with triple therapy of IFN-β1b, and antivirals lopinavir-ritonavir and ribavirin [87]. Similarly, IL-6 blockade could attenuate immune cell mediated pathology, which would also include regulation of NK cells. To truly understand the implications of NK cell therapy in COVID-19 pathology or protection, it would be highly beneficial to use animal models for studying the effect of NK cell depletion and activation in SARS-CoV-2 infections. Overall, more clinical and preclinical data is necessary to take the right steps towards curbing the pandemic and bolstering a future plan in case of recurring SARS-CoV-2 infections.

## Conclusions

The rising death toll of the pandemic COVID-19 has brought the global scientific community to the frontlines of the battle against SARS-CoV-2. Researchers have been mostly focusing on myeloid cells, neutralizing antibodies, and T cells in SARS-CoV-2 infection while an important antiviral defense system, the NK cells, remain understudied. The role of NK cells in other viral infections and tumors have been well established that NK cell–based therapeutics, including CAR-NK cells, activated NK cells, NK cell exhaustion blocking antibodies, and off the line NK cell therapy, are currently in clinical trials for COVID-19. However, there could be serious adverse effects (particularly, overt activation and tissue pathology) associated with these therapies, and the negligible number of studies investigating the pathogenic role of NK cells in COVID-19 do not provide a clear proof of protection versus pathology in infected patients. With the overarching goal of a COVID-19 vaccine and/or immunotherapeutics, a deeper understanding of COVID-19 pathophysiology, the immune evasive strategies employed by the virus, and the interplay of immune cells under the influence of the cytokine storm is desperately needed. In addition, factors such as the type of therapy, timing of therapeutic interventions, disease severity and associated comorbidities, will ultimately influence whether NK cells act as friends or foes in this race to win over the pandemic.
